# Ambiguous allele combinations in HLA Class I and Class II sequence-based typing: when precise nucleotide sequencing leads to imprecise allele identification

**DOI:** 10.1186/1479-5876-2-30

**Published:** 2004-09-13

**Authors:** Sharon D Adams, Kathleen C Barracchini, Deborah Chen, FuMeei Robbins, Lu Wang, Paula Larsen, Robert Luhm, David F Stroncek

**Affiliations:** 1 HLA Laboratory, Department of Transfusion Medicine, National Institutes of Health, Warren G. Magnuson Clinical Center, Bethesda, Maryland USA; 2 DYNAL Biotech, Brown Deer, Wisconsin USA

**Keywords:** HLA, Histocompatibility, genotyping

## Abstract

Sequence-based typing (SBT) is one of the most comprehensive methods utilized for HLA typing. However, one of the inherent problems with this typing method is the interpretation of ambiguous allele combinations which occur when two or more different allele combinations produce identical sequences. The purpose of this study is to investigate the probability of this occurrence. We performed HLA-A,-B SBT for Exons 2 and 3 on 676 donors. Samples were analyzed with a capillary sequencer. The racial distribution of the donors was as follows: 615-Caucasian, 13-Asian, 23-African American, 17-Hispanic and 8-Unknown. 672 donors were analyzed for HLA-A locus ambiguities and 666 donors were analyzed for HLA-B locus ambiguities. At the HLA-A locus a total of 548 total ambiguous allele combinations were identified (548/1344 = 41%). Most (278/548 = 51%) of these ambiguities were due to the fact that Exon 4 analysis was not performed. At the HLA-B locus 322 total ambiguous allele combinations were found (322/1332 = 24%). The HLA-B*07/08/15/27/35/44 antigens, common in Caucasians, produced a large portion of the ambiguities (279/322 = 87%). A large portion of HLA-A and B ambiguous allele combinations can be addressed by utilizing a group-specific primary amplification approach to produce an unambiguous homozygous sequence. Therefore, although the prevalence of ambiguous allele combinations is high, if the resolution of these ambiguities is clinically warranted, methods exist to compensate for this problem.

## Introduction

The precise identification of HLA Class I and Class II alleles is critical for successful hematopoietic progenitor transplants, the development of peptide based viral and cancer vaccines, and investigating immune response [1,2]. DNA sequencing is one of the most comprehensive methods available for HLA typing. Sequence-based typing (SBT) involves PCR amplification of specific coding regions of HLA genes and sequencing of the amplicons [3,4]. SBT allows for a detailed interpretation of HLA alleles by comparing nucleotide sequences of the polymorphic, and sometimes conserved, regions of the HLA gene to a database of possible allelic combinations. While SBT permits the highest resolution of genotypes, like all typing methods, it has limitations. One of the inherent problems when using SBT is the interpretation of ambiguous allele combinations which can occur for several reasons [5-7].

There exist two main types of ambiguous typing results obtained with SBT. The first is when a heterozygous sequence can be explained by more than one possible pair of alleles within the region analyzed. The second exists when alleles are defined by a polymorphism outside the region analyzed. In addition to these two situations, a third type of ambiguity arises when an allele has an incomplete sequence in the region analyzed.

The prevalence of ambiguities in HLA typing relates to the nature of polymorphisms which exists in the sequence of the major histocompatibility complex (MHC) Class I and Class II genes. The majority of polymorphisms that distinguish one MHC allele from another are oftentimes due to gene conversion, recombination and exon shuffling events. Due to this, polymorphic motifs at given positions are generally shared among several alleles.

**Figure 1:**
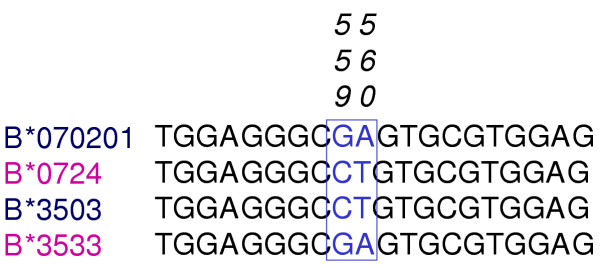
Two different HLA-B allele combinations that yield identical sequence based-typing results. The amplification and sequencing of exon 3 of HLA Class I from a HLA-B*070201/B*0724 subject and a HLA-B*3503/3533 subject produce the same results. Both G and C are detected at nucleotide 559 and both A and T at nucleotide 560.

Sequence-based typing involves PCR amplification and sequencing of specific HLA exons, which are known to be polymorphic, from genomic DNA. For each HLA locus both alleles are amplified and sequenced; therefore, it is not always possible to determine exactly which two alleles were responsible for sequence results. For example two or more different allele combinations can combine to produce identical sequences due to the heterozygous base pair combinations, the first type of ambiguity. More specifically, in the Class I region, HLA-B*070201, 3503 would have the same nucleotide sequence as HLA-B*0724, 3533 in positions 559 and 560 (Figure 1). In this example, the SBT produces a heterozygous base pair combination at positions 559 and 560 with an international union of biochemists (IUB) designation of K(G+C)W(A+T). Therefore, the interpretation to the high resolution level can not be made because it is not known which allele combination is correct.

The second type of ambiguity relates to defining a polymorphism outside the region analyzed. For example, many HLA-A alleles are defined by a polymorphism located in Exon 4 (Table 3). Traditionally, for Class I typing most laboratories only sequence Exon 2 and Exon 3; for Class II typing most laboratories only sequence Exon 2. This approach has been the standard due to the functional relevance of this region which defines the peptide groove of Class I and Class II molecules, respectively. However, some Class I alleles have identical sequences across Exons 2 and 3. To resolve these alleles it is necessary to analyze the gene at the region where they differ. As DNA sequencing has become easier and more widely applied to defining HLA alleles, additional polymorphisms have been found in other exons, and also in the introns.

**Table 3:** Specific A locus sequence-based typing ambiguities discovered during this study and the reason that the ambiguity occurs.

1	A*010101/04N	0104N is resolved as a C insertion at bp 628 in exon 4.
2	A*2402/09N/11N	24020101 is unresolvable from 24020102L. 2409N is resolved in exon 4 at bp 742 by a T substitution. The base change responsible for 2409N creates a premature termination on exon 4. 2411N is the result of the same insertion at 0104N.
3	A*010101/04N, 0201/09/43N or 0236, 3604	0209 is resolved as an A substitution in exon 4 at bp 779. 0243N is resolved as a C insertion at bp 780. 0236 and 3604 are not defined in exon 4.
4	A*020101/43N, 030101 or 0226, 0307 or 0234, 0308	0226 and 0308 are not defined in exon 4. As a result resolution can not be determined.
5	A*0201/09/43N, 2301/07N or 0236, 2304	The 02 alleles are resolved in exon 4 as described above. 2307 N is resolved by a C insert at bp 628 in exon 4. Neither 0236 nor 2304 are defined in exon 4.
6	A*020101/09/43N, 240201/09N/11N or 0212, 2413 or 0236, 24031/33	The 02 and 24 alleles are resolved in exon 4 as described above. Alleles 2413 and 0236 are not defined in exon 4.
7	A*0201/09, 2501 or 0206, 2503	The 02 alleles are resolved in exon 4 as described above. 2503 is not defined in exon 2.
8	A*030101, 240201/09N/11N or 0308, 2407	The 24 alleles are resolved in exon 4 as described above. 0308 is not defined in exon 4.
9	A*030101/2501 or 0308/2502 or 3204/6601	0308, 2502, and 3204 are not defined in exon 4.
10	A*A*030101, 2601 or 0308, 2613	0308 and 2613 are not defined in exon 4.
11	A*240201/09N/11N, 2601 or 2406, 2608	The 24 alleles are resolved in exon 4 as described above. 2406 is undefined in exon 4.
12	A*240201/09N/11N, 2902 or 24031, 2903	The 24 alleles are resolved in exon 4 as described above. 24020101, 2902 and 240301, 2903 are unresolved.
13	A*020101/09/20/43N, 310102/3102	3102 is not defined in exon 4.
14	A*240201/09N/11N, 3201 or 2432, 3203	Both 2432 and 3203 are not defined in exon 4.
15	A*240201/09N/11N, 680102/11N or 2406, 6809 or 2407, 680301	680102 and 6811N are not resolvable. 2406 and 6809 are not defined in exon 4. Exon 4 would resolve 24020101 from 2409N and 2411N. 24020101, 680102/11N is unresolvable from 2407, 680301.
16	A*2502, 7401 or 2502, 7402 or 3201, 6601	7402 is not defined in exon 4. 2502, 7401 is unresolvable from 3201, 6601.

Finally, an ambiguity may be due to incomplete sequence information, because not all alleles have been sequenced for the same exons. For some alleles the entire sequence is not known in the region that is amplified. For example, A*010101 has been sequenced from Exon 1 through Exon 8, but A*010102 has been sequenced only in Exon 2 and Exon 3. Numerous ambiguities arise due to an incomplete sequence in Exon 4. The minimum requirements for submission of new sequences into reference databases of HLA sequences are the sequencing of Exon 2 and Exon 3 for Class I and Exon 2 for Class II.

The relevance of completely identifying the polymorphisms found by SBT needs consideration. In clinical respects, it may not always be necessary to resolve ambiguities that involve a silent non-coding polymorphism and/or an intron polymorphism. Exceptions will exist to this situation where the polymorphism negates or impairs expression (e.g. A*24020102L or B*15010102N – both are due to an intron polymorphism). However, for investigations of genetic inheritance or disease association, the definition of all polymorphisms may be significant.

The purpose of this study was to summarize the incidence, nature, and cause of ambiguous HLA SBT results. This represents an important step toward developing strategies to reduce or eliminate this problem.

## Methods

### DNA Isolation

Genomic DNA was isolated from peripheral blood using the Gentra PUREGENE^® ^isolation kit (Gentra Systems, Minneapolis, MN, U.S.A). The DNA was resuspended in Tris HCl buffer (pH 8.5) and the concentration was measured using a Pharmacia Gene Quant II Spectrophotometer. The DNA was then stored at -70°C until testing.

### Sequence-Based Typing

The primary PCR amplification reaction consists of a 1.5 kb reaction encompassing exon 1 through intron 3 of the HLA region. All reagents necessary for primary amplification and sequencing are supplied in the HLA-A or HLA-B AlleleSEQR Sequenced Based Typing Kits (Forensic Analytical, Hayward, CA, U.S.A.). The primary amplification PCR products were purified from excess primers, dNTPs, and genomic DNA using ExoSAP-IT (Amersham Life Science, Cleveland, OH, U.S.A.) Each template was sequenced in the forward and reverse sequence orientation for exon 2 and Exon 3 according to protocols supplied with the SBT kit. Excess dye terminators were removed from the sequencing products utilizing an ethanol precipitation method with absolute ethanol. The reaction products were reconstituted with 15 μl of Hi-Di™ Formamide (PE Applied Biosystems/Perkin-Elmer, Foster City, CA, U.S.A.) and analyzed on the ABI Prism^® ^3700 DNA Analyzer with Dye Set file: Z and mobility file: DT3700POP6 {ET}.

## Results

Sequence based typing analysis of HLA-A and B alleles was performed on a population of 676 normal donors. The racial distribution of the subjects studied was: 615 Caucasian, 13 Asian, 23 African American, 17 Hispanic and 8 Unknown. 672 of the 676 subjects were analyzed for the presence of HLA-A locus ambiguities and 666 were analyzed for HLA-B locus ambiguities. Each allele was counted separately in this analysis in order to determine the total percentage of ambiguous allele combinations. Four new potential alleles were found.

At the HLA-A locus a total of 548 ambiguous allele combinations were found. This represented 41% of all HLA-A alleles (548 of 1344) (Table 1). Approximately half, 51% (278 of 548) of these ambiguities were due to the fact that Exon 4 analysis was not performed (Table 2 and Table 3). HLA-A*01 and HLA-A*24 are very prevalent alleles and most ambiguities involving these alleles could be resolved by performing Exon 4 analysis. For example the ambiguity most prevalent for HLA-A*01 in this study was HLA-A*0101/0104N. The sequences of these two alleles are identical across Exons 2 and 3; the difference between these two alleles occurs at position 627insC, which is located in Exon 4.

**Table 1:** Prevalence of ambiguous sequence-based typing allele combinations among 672 people analyzed at the HLA-A locus and 666 analyzed at the HLA-B locus

**Allele**	**Occurrence**	**Allele**	**Occurrence**
A*01	174	B*07	51
A*02	162	B*08	56
A*03	43	B*13	2
A*23	2	B*14	3
A*24	104	B*15	26
A*25	5	B*18	17
A*26	26	B*27	37
A*29	3	B*35	34
A*31	4	B*39	3
A*32	5	B*40	31
A*66	1	B*44	44
A*68	16	B*51	14
A*74	3	B*52	1
		B*55	2
		B*58	1
**TOTAL**	**548**	**TOTAL**	**322**

**Table 2:** Nature and Resolution of HLA-A and -B Allele Ambiguities

	**HLA-A Locus**	**HLA-B Locus**
Portion of alleles with ambiguities	41%	24%
Most frequently involved alleles	A*01, 02, and 24	B*7, 8, 15, 35, and 44
Methods used to resolve ambiguities	Exon 4 sequencing (51%)	Many different methods
	A*02 subtyping (30%)	

A large portion of HLA-A locus sequence-based typing ambiguities involved HLA-A*02, 30% or 162 of 548. Some of the HLA-A*02 ambiguities can also be resolved via Exon 4 sequencing and most of the other HLA-A*02 ambiguities can be resolved with traditional A*02 molecular subtyping methodologies using sequence specific primers or sequence specific probes.

Not all HLA-A ambiguous allele combinations can be resolved as simply as those involving A*01, A*024 and A*02. Most of the remaining 19% (108 of 548) of the HLA-A ambiguities cannot be resolved with Exon 4 analysis (Table 3).

Review of the HLA-B locus results revealed 322 ambiguous allele combinations among the 1332 total HLA-B alleles (24%). Antigens HLA-B*07/08/15/27/35/44, common in Caucasians, produced the largest portion of the ambiguities (279 of 322 or 87%). Each of these ambiguities had an independent reason for occurring. Table 4 lists some of the more common B locus ambiguities seen in this study. The reason for each ambiguity is variable; however a large portion of the ambiguities are related to cis/trans allele combinations.

**Table 4:** Comparison of B locus ambiguities seen with the standard single tube amplification sequence-based typing method utilized in this study and those expected to be seen when utilizing a new two tube sequence-based typing method.

**Ambiguities using a single tube amplification**
B*270502 or 270502/13 or 2713
B*070201, 0801 or 0705/06, 0807
B*070201, 1402 or 0726, 1403
B*0702, 150101 or 0707, 1507 or 0709, 1563
B*070201, 180101/17N or 0707, 1814 or 0726, 1813
B*07021, 400101/0102 or 0705/06, 4033
B*070201, 440201/19N/27 or 0720, 4416 or 0724, 4421
B*0801, 180101/17N or 0804, 1807 or 0812, 1814
B*0801, 400101/0102 or 0804, 4007
B*0801, 440201/19N/27 or 0802, 4409
B*150101/15, 3503/13
B*1503, 3501 or 1529, 3528
B*3501, 400101/0102 or 3520, 4007
B*350101/40N/42, 4402/19N/27 or 3510, 4412
B*400101, 4402 or 400102, 4402 or 4042, 4414
B*4001, 510101 or 4007, 5107
**Ambiguities reduced by 56% if Two Tube Group Amplification is Used**
Remaining ambiguities:
B*070201, 180101/17N or 0707, 1814 or 0726, 1813
B*070201, 440201/19N/27 or 0720, 4416 or 0724, 4421
B*0801, 180101/17N or 0804, 1807 or 0812, 1814
B*0801, 440201/19N/27 or 0802, 4409
B*150101/15, 3503/13
B*1503, 3501 or 1529, 3528
B*3501, 400101/0102 or 3520, 4007
B*400101, 4402 or 400102, 4402 or 4042, 4414
B*4001, 510101 or 4007, 5107

## Discussion

While SBT provides the best available typing of HLA-A and B antigens, it is limited by sequence results that don't allow the precise identification of alleles. We found that 41% of HLA-A alleles and 24% of HLA-B alleles were ambiguously typed. The ambiguities involve some of the most frequent HLA-A and HLA-B antigens: A*01, A*02, A*24, B*07, B*08, B*15, B*27, B*35, and B*44. However, ambiguous allele combinations occur in all loci tested in HLA. The IMGT/HLA Sequence Database  maintains an updated listing of all ambiguous possibilities [5,8].

The need to initiate additional testing to clarify ambiguous allele combinations must consider whether it is practical to obtain the information and if the information is useful and valuable. The clinical need for the highest resolution HLA typing possible is an important variable that must be considered. When typing is performed for cancer and viral vaccine development studies, high resolution allele data may be necessary to determine if a subject has an HLA type that is appropriate for a study. Utilization of high resolution data may also have implications for hematopoietic progenitor cell transplantation. Transplants involving partially mismatched or unrelated donor-recipient pairs require a higher resolution typing, but those involving HLA identical siblings may not.

If it is necessary to resolve an ambiguous typing, a variety of different methods can be used. If the ambiguity is due to an allele that has not been completely sequenced or because the ambiguity is outside the region amplified by the SBT assay, the resolution is dependent on the nature and complexity of the ambiguity. Traditionally, for Class I sequencing purposes most laboratories have performed Exon 2 and Exon 3 analysis alone and for Class II sequencing only Exon 2 analysis. Many of the ambiguities can be resolved by sequencing Exon 4. In fact, in this study the largest portion of the typing ambiguities can be resolved by sequencing exon 4. However, many polymorphisms in exon 4 have no functional significance, so it may not be worthwhile resolving most ambiguities involving exon 4. The requirement for the analysis of Exon 4 to reduce the incidence of typing ambiguity has now been realized by commercial kit manufacturers. Both Celera Diagnostics (Alameda, CA) and Forensic Analytical/Atria Genetics (South San Francisco, CA) now include reagents for analysis of exon 4 in the HLA-A and -B kits. As the use of SBT increases, more data may become available from non-traditional exons in addition to those that have been traditionally sequenced and the number of ambiguities due to unknown sequences will decrease.

If the ambiguity is due to an identical heterozygote sequence, as shown in figure 1, the ambiguous allele combinations can sometimes be addressed by utilizing a group-specific primary amplification approach. In this approach each allele is amplified separately by using group specific primers for the alleles in question. A homologous sequence for each separate allele can then be obtained by sequencing the product of the group specific amplifications. Currently, there are commercially available kits (Forensic Analytical, Hayward, CA) for group specific amplification of the B locus. These kits allow the primary amplification of a specific group. Upon discovery of a particular ambiguity, a group specific amplification is done to separate out the allele pair. The resultant sequence will be homozygous for each allele in question. Another method, which will reduce the number of ambiguities in the B locus, is the utilization of a two tube group amplification approach (DYNAL Biotech, Brown Deer, WI). This method allows for resolution of ambiguities by taking into account the cis/trans allele combinations which result from simultaneous nucleotide incorporation for DNA templates being sequenced. This method allows for separation of ambiguities when the ambiguity has arisen due to a cis/trans situation. Ambiguities utilizing this method are reduced by 56%. (Table 4) Another method for separation of alleles is Haploprep™. (Genovision, Philadelphia, PA). Haploprep™ physically separates a diploid sample into its haploid components. Once the haplotypes are separated, routine HLA typing methods can be performed to determine the alleles. This laboratory is currently conducting studies to determine the efficacy of this product. Several of the remaining HLA loci ambiguities can be managed utilizing in-house custom group specific primary amplification mixes.

Other methods which have been utilized to produce a homologous sequence include cloning, reference strand conformational analysis (RSCA), Pyrosequencing™ and denaturing high-performance liquid chromatography (DHPLC) [9-11]. Pyrosequencing™ is being explored by this laboratory and results at this time are preliminary. This method relies on the identification of a correct dispensation order of nucleotides during the Pyrosequencing™ process. Each ambiguity would require a separate dispensation order to be determined due to the unique nature of each ambiguity. The initial setup of this technology may be cumbersome; however, once established it may become very streamlined due to the availability of data on different ambiguous allele combinations. Each one of these methods has advantages and concerns which must be thoroughly investigated by the laboratory. Some, not all, ambiguous allele combinations produced by having identical heterozygote combinations can be resolved utilizing traditional sequence specific primers (SSP) or sequence specific oligonucleotide probes (SSOP). This may be a more viable approach for laboratories if they are already performing one of these technologies.

In conclusion, although the prevalence of ambiguous allele combinations is high, methods exist to compensate for this problem. As the HLA field continues the discovery of new alleles, alternative approaches to discerning ambiguous allele combinations will need to be investigated in order to reduce the ever-growing number of ambiguities.
